# Efficacy of hormonal and mental treatments with MMPI in FtM individuals: cross-sectional and longitudinal studies

**DOI:** 10.1186/s12888-017-1423-y

**Published:** 2017-07-17

**Authors:** Hiroyuki Oda, Toshihiko Kinoshita

**Affiliations:** Department of Neuropsychiatry, Kansai Medical University, 10-15 Fumizono-cho, Moriguchi City, Osaka, 570-8506 Japan

**Keywords:** Gender Dysphoria, Female-to-male individuals, Minnesota Multiphasic personality inventory (MMPI), Cross-sex hormonal treatment, Psychotherapy

## Abstract

**Background:**

Cross-sex hormone treatment (CSHT) is an important option for gender dysphoria (GD) individuals to improve the quality of life. However, in Japan, sex reassignment surgery (SRS) and CSHT for GD had been discontinued until 1998 (over 30 years). After resumption, the number of GD individuals wishing treatment rapidly increased. On the other hand, the number of medical institutions available for evaluation was limited. For this reason, hormonal treatment has been administered to GD individuals requiring the prompt start of CSHT in the absence of mental health assessment by specialists. In this study, we examined the efficacy of CSHT and psychotherapy.

**Methods:**

The participants were 155 female-to-male (FtM) individuals who consulted our gender identity clinic, and were definitively diagnosed. A cross-sectional study was conducted by dividing them into two groups: groups with and without CSHT on the initial consultation (Group CSHT: *n* = 53, Group no-CSHT: *n* = 102). In all participants, Minnesota Multiphasic Personality Inventory (MMPI) and blood hormone tests were performed on the initial consultation. In addition, CSHT was combined with psychotherapy for a specific period in Group no-CSHT, and FtM individuals in whom an additional MMPI test could be conducted (Group combined treatment (CT), *n* = 14) were enrolled in a longitudinal study.

**Results:**

In the cross-sectional study, there was no significant difference on the MMPI test. In the longitudinal study, there were improvements in the clinical scales other than the Mf scale on the MMPI test. In Group CT, the D, Sc, and Si scale scores on the initial consultation were significantly higher than in Group CSHT. However, there was no clinical scale with a significantly higher value after the start of treatment. The Pd scale score was significantly lower.

**Conclusions:**

CSHT improved mental health. Psychotherapy-combined CSHT may further improve it.

**Trial registration:**

The study was reviewed and approved by the Ethics Committee of Kansai Medical University (A comprehensive treatment for gender dysphoria: No. 0314 registered date 10th December 2003), and was approved at UMIN000028102 on 6th July 2017 as retrospectively registered.

## Background

In Japan, gender dysphoria (GD) refers to a condition in which biological sex is not consistent with self-consciousness or self-recognition of gender [1]. In the International Statistical Classification of Diseases and Related Health Problems (ICD-10) [2] and Diagnostic and Statistical Manual of Mental Disorders, fifth edition (DSM-5) [3], diagnostic criteria are defined, and GD is internationally known. In 2013, the American Psychiatric Association changed the concept of diagnosis. As a result, although GD is included in DSM-5, GD was no longer Gender Identity “Disorder”. Recently, GD is not always related to severe comorbid psychiatric findings, and is better characterized by experience and discomfort associated with gender dysphoria.

On the other hand, formal epidemiologic studies on the incidence and prevalence of transsexualism specifically or transgender and gender nonconforming identities in general have not been conducted, and efforts to achieve realistic estimates are fraught with enormous difficulties [4, 5]. And, for various reasons, researchers who have studied incidence and prevalence have tended to focus on the most easily counted subgroup of gender nonconforming individuals: transsexual individuals who experience gender dysphoria and who present for gender-transition-related care at specialist gender identity clinics [6]. According to the report, the rate of male-to-female (MtF) individuals was 1/11,900 to 45,000, and that of female-to-male (FtM) individuals was 1/30,400 to 200,000 [7]. And some scholars have suggested that the prevalence is much higher, depending on the methodology used in the research [8].

In Japan, a survey was performed by the Special Committee for Gender Dysphoria of the Japanese Society of Psychiatry and Neurology in 2013, and the number of GD individuals who consulted a medical institution, complaining of GD, was estimated to be approximately 17,000. This number corresponds to 1/7000 in Japan. It is higher than the prevalence rate described by De Cuyperera [6]. However considering indications by Olyslager & Conway [7], the actual number may be greater. Furthermore, the FtM-to-MtF ratio in Japan is 2.2:1 [3]. This ratio is different compared to some western country.

Concerning medical practice for GD, there are specific circumstances in Japan. In 1969, sex reassignment surgery (SRS) for MtF individuals was evaluated as guilty for the following reasons: evaluation for diagnosis was not performed, and informed consent regarding SRS was not obtained. However, SRS was misunderstood as violating the laws. As a result, not only SRS but also physical treatment, including CSHT, was officially avoided. In 1997, the “Guidelines for the Diagnosis and Treatment of GD” were published by the Japanese Society of Psychiatry and Neurology [9]. Strict criteria for evaluation and physical treatment indication were established so that a judgment of guilty might not be given again. In 1998, SRS was performed according to the guidelines. The guidelines in Japan were prepared with reference to the standards of care for the health of transsexual, transgender, and gender nonconforming people, 7th edition (SOC7) [4]. Currently, the 4th version has been announced [10].

Although medical practice for GD, which had been discontinued over approximately 30 years, was resumed, there are few physicians who can evaluate GD and make a definitive diagnosis or such medical institutions. GD individuals had to wait for 6 to 12 months until consultation at a gender identity clinic. To GD individuals requiring the prompt start of CSHT, hormonal treatment has been administered based on gynecologists’ or urologists’ evaluation at local clinics in the absence of assessment by mental health specialists. As a result, GD individuals who consulted our gender identity clinic consisted of those who had not been evaluated and those in whom hormonal treatment was started without evaluation.

Recently, several studies in other countries evaluated the effects of hormonal treatment on FtM. The efficacy of CSHT was reviewed by Colizzi and Costa [11]. Concerning FtM, some studies assessed the quality of life (QOL) using the 36-Item Short Form Health Survey (SF-36) [12, 13], and others using the Social Anxiety and Distress Scale (SADS) and Hospital Anxiety and Depression Scale (HADS) [14] or using the Zung Self-rating Anxiety Scale (SAS), Zung Self-rating Depression Scale (SDS), and Symptom Checklist-90-R (SCL-90-R)(longitudinal study) [15] indicated that hormonal treatment seemed to have a positive effect on GD individuals’ mental health. This remains controversial, but many investigators supported the effects of hormonal treatment.

The Minnesota Multiphasic Personality Inventory (MMPI) involves many clinical scales, facilitating many-sided personality assessment. Therefore, it has been used for the psychological assessment of GD [16–26]. Several studies investigated CSHT using the MMPI [27–31]. However, no longitudinal study has compared changes before and after CSHT in FtM individuals using the MMPI.

In this study, the effects of assessment-free CSHT were initially examined. Subsequently, the effects of CSHT with evaluation and psychotherapy were investigated. As the number of MtF individuals was small, FtM individuals were adopted as participants. The participants were divided into two groups based on the presence or absence of CSHT, and its effects were evaluated based on the MMPI test results and blood hormone levels.

The protocol of this study was approved by the Ethics Review Board of Kansai Medical University. Written informed consent was obtained from all FtM individuals on the initial consultation after explaining that information on medical and psychological examinations may be published in academic journals.

## Methods

Since 1999, Kansai Medical University Medical Center has been responsible for GD treatment. According to the guidelines in Japan, comprehensive treatment has been performed by a medical team consisting of psychiatrists, urologists, gynecologists, plastic surgeons, psychiatric social workers, clinical psychologists, and clerical employees [31].

The participants of this study were 183 FtM individuals who were definitively diagnosed at our gender identity clinic before the end of December 2008. The participants comprised our series. Informed consent regarding study participation was obtained from all participants. For diagnosis, it was confirmed that the participants met the diagnostic criteria for GID presented in the DSM-IV-TR [32], and that no participant denied the essential gender identity or wished to undergo SRS due to mental disorder such as schizophrenia. Definitive diagnoses were made by two psychiatrists based on a consistency in the results of evaluation.

Of the 183 FtM individuals, we analyzed 155 (mean age: 25.6 ± 5.9 years [15–43 years]), excluding 28 in whom hormone levels were measured before July 1, 2004 because hormone-measuring methods were changed on July 1, 2004. The 155 participants had not undergone assessment or psychotherapy in other hospitals.

They were divided into two groups: FtM individuals who had received CSHT before the initial consultation at the gender identity clinic (Group CSHT) and those who had not received it (Group no-CSHT). In addition, in the latter, FtM individuals in whom CSHT was started in combination with psychotherapy (combined treatment) based on the results of evaluation after the start of outpatient care were assigned to Group CT.

Of the 155 FtM individuals, 53 comprised Group CSHT (mean age: 26.8 ± 6.0 years [17–43 years]), 102 comprised Group no-CSHT (mean age: 24.9 ± 5.8 years [15–41 years]) (Table 1), and 14 comprised Group CT (mean age: 23.9 ± 4.1 years [18–32 years]) (Table 2). There were no significant differences in the mean age among the 3 groups. Furthermore, there were no marked differences in the school period or working state based on the information obtained on consultations.

**Table 1:** Classification (cross-sectional study)

		First Visit		
		Psychotherapy	CSHT	MMPI
CSHT	*n* = 53	−	+	+
no-CSHT	*n* = 102	−	−	+

**Table 2:** Classification (longitudinal study)

			Psychotherapy	CSHT	MMPI
CT	*n* = 14	First Visit	−	−	+
		After Treatment	+	+	+

We conducted several psychological tests early after the initial consultation to assist a diagnosis and evaluate adaptability to physical treatment. In particular, the MMPI is a personality test with a questionnaire consisting of 383 items. This test requires approximately 1 h, but the current mental state can be simply evaluated using 10 clinical scales (hypochondriasis (Hs), depression (D), hysteria (Hy), psychiatric disease qualitative deviation (Pd), masculinity/femininity (Mf), paranoia (Pa), psychasthenia (Pt), schizophrenia (Sc), hypomania (Ma), and social introversion (Si) scales). Therefore, this test may also be important to obtain suggestions regarding the indication of treatment. Furthermore, the clinical scales include a scale regarding male and female sex (Mf scale), which is characteristic. Since the 1970’s, this scale has been used to evaluate the psychological properties of GD [33].

In addition to the MMPI test, the blood levels of testosterone and estradiol were measured as blood hormone levels on the initial consultation. In addition, in Group CT, the second MMPI test was conducted after performing CSHT for a specific period (519 ± 365 days) in parallel with psychotherapy.

To confirm the effects of previous CSHT, the blood levels of testosterone and estradiol on the initial consultation in Group CSHT were compared with those in Group no-CSHT. In addition, to evaluate the relationship between the hormone levels and mental state, we analyzed the relationship between the clinical scales of the MMPI and blood testosterone and estradiol levels in the same groups using Pearson’s test.

To evaluate the effects of CSHT, the MMPI clinical scale scores on the initial consultation were compared between Groups CSHT and no-CSHT using the Mann-Whitney U-test.

To evaluate the effects of starting CSHT during psychotherapy following assessment on the mental state, the MMPI clinical scale scores after a specific period of CSHT in Group CT were compared with those on the initial consultation using Wilcoxon’s coded rank test.

To examine the effects of CSHT with respect to the presence or absence of psychotherapy, the MMPI clinical scale scores on the initial consultation in Group CT were compared with those in Group CSHT using the Mann-Whitney U-test. In addition, the scores after the start of CSHT in Group CT (on the second MMPI test) were compared with those in Group CSHT.

Essentially, participants in whom hormonal treatment was started after the initial consultation should be randomly divided into two groups: CT and non-psychotherapy-combined groups, and the results should be compared. However, according to the guidelines in Japan, CSHT cannot be started in the absence of psychotherapy. Therefore, this study involved Group CSHT, in which CSHT had been performed before the initial consultation in the absence of psychotherapy. (Psychotherapy described in the guidelines in Japan refers to mental support, a review of real life experience (RLE), and assistance to improve the QOL in a new life).

## Results

**Fig. 1:**
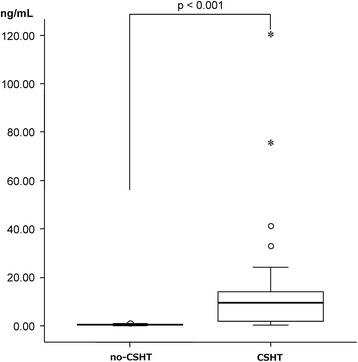
Intergroup comparison of testosterone levels. Normal range (Female: 0.13–0.69, Male: 2.07–7.61)

**Fig. 2:**
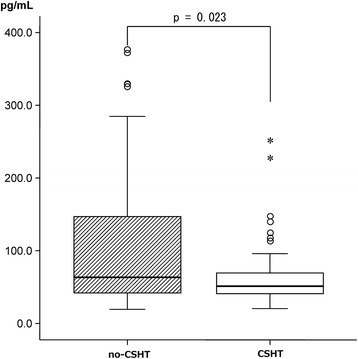
Intergroup comparison of estradiol levels. Normal range (Follicular phase: early 20–85, late 25–350, Ovulation phase 50–550, Luteal phase 45–300, Post menopause 21 or lower, Men 15–35)

With respect to hormone levels, the blood testosterone level in Group CSHT was significantly higher (Fig. 1), and the blood estradiol level was significantly lower (Fig. 2). Concerning the correlation between the hormone levels and MMPI clinical scales (Table 3), the blood testosterone level was not correlated with any clinical scale in Groups CSHT or no-CSHT. In Group CSHT, the blood estradiol level was positively correlated with the D and Pt scales. In Group no-CSHT, it was not correlated with any clinical scale.

**Table 3:** Correlations between testosterone/estradiol levels and the MMPI clinical scales

		Hs	D	Hy	Pd	Mf	Pa	Pt	Sc	Ma	Si
Testosterone levels	CSHT	0.821	0.803	0.424	0.532	0.947	0.665	0.917	0.789	0.435	0.453
	no-CSHT	0.237	0.158	0.408	0.707	0.768	0.387	0.322	0.420	0.870	0.584
Estradiol levels	CSHT	0.079	0.337	0.144	0.377	0.549	0.067	0.698	0.188	0.542	0.987
	no-CSHT	0.254	0.037*	0.064	0.062	0.962	0.241	0.023*	0.268	0.475	0.286
			(0.287)					(0.313)			

^*^ *p* < 0.05, () Pearson Correlation Coefficient

**Fig. 3:**
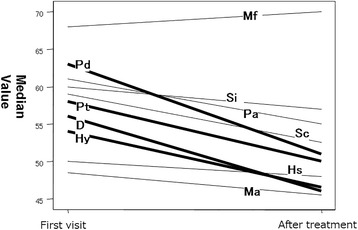
Median value of first visit and after treatment. 519 ± 365 days: No. of days after initiation of hormonal treatment

There were no significant differences in any MMPI clinical scale between Groups CSHT and no-CSHT (Table 4). After the start of hormonal treatment, improvements in the MMPI clinical scale scores, other than the Mf scale score, were achieved in comparison with the values on the initial consultation. In particular, there were significant improvements in the D, Hy, Pd, and Pt scale scores (Table 5) (Fig. 3).

**Table 4:** Intergroup comparisons of the clinical scales

	Hs	D	Hy	Pd	Mf	Pa	Pt	Sc	Ma	Si
Two-sided *p* value	0.270	0.752	0.806	0.383	0.821	0.406	0.902	0.297	0.717	0.983

No significant differences were observed

**Table 5:** Intergroup comparisons of the clinical scales

	Hs	D	Hy	Pd	Mf	Pa	Pt	Sc	Ma	Si
Two-sided *p* value	0.124	0.013*	0.006*	0.017*	0.861	0.071	0.023*	0.050	0.328	0.054

*p < 0.05

When comparing the results on the initial consultation in Group CT with those in Group CSHT (Table 6), the D, Sc, and Si scale scores were significantly higher. When comparing the results of the second MMPI test in Group CT with those in Group CSHT (Table 7), the Pd scale score was significantly lower.

**Table 6:** Intergroup comparisons of the clinical scales

	Hs	D	Hy	Pd	Mf	Pa	Pt	Sc	Ma	Si
Two-sided *p* value	0.400	0.034*	0.342	0.487	0.699	0.058	0.115	0.046*	0.430	0.006*

*p < 0.05

**Table 7:** Intergroup comparisons of the clinical scales

	Hs	D	Hy	Pd	Mf	Pa	Pt	Sc	Ma	Si
Two-sided *p* value	0.688	0.562	0.066	0.010*	0.804	0.474	0.276	0.932	0.142	0.208

*p < 0.05

## Discussion

CSHT is technically easier than surgery, and its cost is relatively inexpensive. For this reason, CSHT for FtM individuals is routinely performed to relieve psychiatric symptoms associated with gender dysphoria-related mental distress and acquire a better mental well-being. Therefore, its effects on the mental state should be particularly evaluated in detail.

We initially investigated the blood levels of hormones to evaluate the presence or absence of CSHT, and confirmed that the influence of hormone agents was strongly reflected in Group CSHT. The blood testosterone level was significantly higher (Fig. 1), and the estradiol level was significantly lower (Fig. 2). In particular, the median blood testosterone level exceeded the upper limit of the reference range in males, and the first quartile was twice as high as the upper limit of the reference range in males. Based on these results, we confirmed that CSHT had been performed in Group CSHT; an excessive dose of CSH may have been administered to some participants. Thus, there were marked CSHT-related changes in the hormone levels, but there were no significant differences in the MMPI clinical scale scores between Group CSHT and Group no-CSHT (Table 4). Concerning the correlation between the level of each hormone and mental state, the estradiol level was weakly correlated with the D and Pt scales, and there was no marked correlation (Table 3).

Previous cross-sectional [12–14, 30, 34–37] and longitudinal [15, 38–41] studies examined the relationship between CSHT and the mental health involving FtM individuals. Some cross-sectional studies [40, 41] reported the significant effects of CSHT in MtF individuals alone. However, in other studies, the significant effects of CSHT were also obtained in FtM individuals. Several studies compared FtM individuals receiving CSHT with those who had not received CSHT [12] or non-GD individuals [13] using the SF-36, and suggested a positive effect of hormone therapy on GD individuals’ QOL. A study evaluated GD individuals before SRS using the Social Self-Esteem Inventory (SSEI), Beck Depression Inventory (BDI), Subjective Quality of Life Analysis (SQUALA), and Global Assessment of Functioning scale (GAF), and reported that hormone therapy was less severe depression symptoms [34]. Another study evaluated GD individuals using the World Health Organization Quality of Life-shorter version (WHOQOL-BREF), and reported that CSHT was linked to a better self-reported QOL in GD [34]. Furthermore, another study compared GD individuals receiving CSHT with those who had not received it using the SADS and HADS, and reported its positive effects on social distress, anxiety, and depression [14]. Another study involving FtM individuals before SRS using the MMPI showed that CSHT improved psychopathology [30].

Several longitudinal studies demonstrated the efficacy of CSHT using the SAS, SDS, SCL-90-R, Structured Clinical Interview for Diagnostic and Statistical Manual of Mental Disorders I (SCID-I) [15], SCL-90-R, Psychosocial questionnaires [38], Dissociative Experiences Scale (DES) [39], blood cortisol level, Perceived Stress Scale (PSS) [40], expectancy list of mood and sexual interest (ELOMS), affect intensity measure (AIM), short anger situation questionnaire (ASQ), affective communication test (ACT), and premenstrual assessment form (PAF) [41].

In this study, there was no significant difference in any MMPI clinical scale score between Groups CSHT and no-CSHT. The results of previous cross-sectional studies suggest the positive effects of CSHT in FtM individuals [12–14, 30, 34, 36]. However, a previous study using the MMPI did not show any significant difference [37]. It was indicated that, when the MMPI clinical scale score was within the normal range, it did not reflect the influence of CSHT [37]. In addition, in Group CSHT, which was analyzed in this study, CSHT was started because FtM individuals could not wait until assessment at a gender identity clinic due to specific circumstances in Japan. CSHT may have improved the mental health from a state in which FtM individuals wished to start CSHT promptly to the level of Group no-CSHT in which FtM individuals could wait for a long period until consultation, thus leading to the absence of a significant difference between Groups CSHT and no-CSHT.

In Group CT, improvements in the clinical scale scores other than the Mf scale score were achieved after CSHT. In particular, there were significant improvements in the D, Hy, Pd, and Pt scale scores. In addition, the D, Sc, and Si scale scores on the initial consultation in Group CT were significantly higher than in Group CSHT, but the Pd scale score was significantly lower after treatment. This suggests that CSHT combined with psychotherapy improves the mental health. Although the efficacy of CSHT was confirmed, CSHT-related apparent masculinization may affect the FtM individuals’ relationship with others. Furthermore, the effects of CSHT are marked in FtM individuals, but it may stimulate aggression [41]. The combination of CSHT and psychotherapy, involving mental support and a review of RLE, as well as continuous assistance to improve the QOL in a new life, may have led to mental stability.

Concerning the Mf scale, Bonierbale et al. reported that the scale score in the CSHT group was significantly lower than in the no-CSHT group [30]. They indicated that CSHT have less need to assert themselves because of their bodies and their perception that their appearance is more in accordance with their stereotype [30]. On the other hand, our study did not show any significant difference between Groups CSHT and no-CSHT or before and after CSHT in Group CT. Briefly, CSHT did not influence the Mf scale score regardless of the presence or absence of psychotherapy. In Japan, SRS is a requirement for changing the sex in the family register; therefore, CSHT reduces body uneasiness, but, socially, the FtM individuals’ gender is female. This may not have contributed to a reduction in the assertion of being male, leading to the results differing from those reported by Bonierbale.

A mental health screening and/or assessment is needed for referral to hormonal treatment for GD [4, 10]. In contrast, psychotherapy – although highly recommended – is not a requirement [4]. In Japan, it is recommended that CSHT should be performed while continuing psychotherapy involving sympathy with psychological/social/physical stress and a review of RLE-related problems at working places/schools [10]. We previously reported that favorable results were obtained when performing CSHT in such a state [42]. The results of this study also suggest that, when performing CSHT in FtM individuals, further improvements may be achieved by evaluating the mental health and combining psychotherapy.

However, our study has a limitation: when performing CSHT, it was impossible to assign the participants to psychotherapy and psychotherapy-free groups and evaluate the results, because CSHT cannot be started in the absence of psychotherapy in accordance with the guidelines in Japan.

## Conclusions

We examined the effects of CSHT and psychotherapy in FtM individuals using the MMPI through cross-sectional and longitudinal studies. In the cross-sectional study, we evaluated the results with respect to the presence or absence of CSHT. However, there were no significant differences in the MMPI clinical scale scores. In the longitudinal study, the combination of CSHT and psychotherapy significantly improved the MMPI clinical scale scores. CSHT improve mental health, and, when combined with psychotherapy, further improvements may be achieved.

## Abbreviations

ACT: Affective communication test
AIM: Affect intensity measure
ASQ: Short anger situation questionnaire
BDI: Beck Depression Inventory
CSHT: Cross-sex hormone treatment
CT: Combined treatment
D: Depression
DES: Dissociative Experiences Scale
DSM-IV-TR: Diagnostic and Statistical Manual of Mental Disorders, text revision
ELOMS: expectancy list of mood and sexual interest
FtM: Female-to-male
GAF: Global Assessment of Functioning scale
GD: Gender dysphoria
GID: Gender identity disorder
HADS: Hospital Anxiety and Depression Scale
Hs: Hypochondriasis
Hy: Hysteria
ICD-10: International Statistical Classification of Diseases and Related Health Problems
Ma: Hypomania
Mf: Masculinity/Femininity
MMPI: Minnesota Multiphasic Personality Inventory
MtF: Male-to-female
Pa: Paranoia
PAF: premenstrual assessment form
Pd: Psychopathic Deviate
PSS: Perceived Stress Scale
Pt: Psychasthenia
SADS: Social Anxiety and Distress Scale
SAS: Zung Self-rating Anxiety Scale
Sc: Schizophrenia
SCID-I: Structured Clinical Interview for Diagnostic and Statistical Manual of Mental Disorders I
SCL-90-R: Symptom Checklist-90-R
SDS: Zung Self-rating Depression Scale
SF-36: 36-Item Short Form Health Survey
Si: Social Introversion
SQUALA: Subjective Quality of Life Analysis
SRS: Sex reassignment surgery
SSEI: Social Self-Esteem Inventory
WHOQOL-BREF: World Health Organization Quality of Life-shorter version
